# Expanding care for perinatal women with depression (EXPONATE): study protocol for a randomized controlled trial of an intervention package for perinatal depression in primary care

**DOI:** 10.1186/s12888-015-0537-3

**Published:** 2015-06-30

**Authors:** Oye Gureje, Bibilola Damilola Oladeji, Ricardo Araya, Alan A. Montgomery, Lola Kola, Laurence Kirmayer, Phyllis Zelkowitz, Danielle Groleau

**Affiliations:** 1 Department of Psychiatry, College of Medicine, University of Ibadan, Ibadan, Nigeria; 2 Centre for Global Mental Health, London School of Hygiene and Tropical Medicine, London, UK; 3 Nottingham Clinical Trials Unit, University of Nottingham, Queen’s Medical Centre, Nottingham, UK; 4 Division of Social and Transcultural Psychiatry, McGill University Culture and Mental Health research unit, Jewish General Hospital, Montréal, Canada

**Keywords:** Perinatal depression, Primary care, Cluster randomized trial

## Abstract

**Background:**

Depression is common among women during perinatal period and is associated with long-term adverse consequences for the mother and infant. In Nigeria, as in many other low- and-middle-income countries (LMIC), perinatal depression usually goes unrecognized and untreated. The aim of EXPONATE is to test the effectiveness and cost-effectiveness of an intervention package for perinatal depression delivered by community midwives in primary maternal care in which physician support and enhanced patient compliance are implemented using mobile phones.

**Methods/Study design:**

A pragmatic two-arm parallel cluster randomized controlled trial was designed. The units of allocation are the primary maternal care clinics. Thirty eligible and consenting clinics were randomized but, due to problems with logistics, 29 eventually participated. Consenting pregnant women with a gestational age between 16 and 28 weeks who screened positive on the Edinburgh Postnatal Depression Scale (EPDS score ≥12), absent psychosis or bipolar disorder, and not actively suicidal were recruited into the trial (N = 686). Midwives in the intervention arm were trained to deliver psychoeducation, problem solving treatment, and parenting skills. Eight weekly sessions were delivered following entry into the study. Further sessions during pregnancy and 6 weeks following childbirth were determined by level of depressive symptoms. Clinical support and supervision, delivered mainly by mobile phone, were provided by general physicians and psychiatrists. Automated text and voice messages, also delivered by mobile phones, were used to facilitate patient compliance with clinic appointments and ‘homework’ tasks. Patients in the control arm received care as usual enhanced by further training of the providers in that arm in the recognition and standard treatment of depression. Assessments are undertaken at baseline, 2 months following recruitment into the study and 3, 6, 9 and 12 months after childbirth. The primary outcome is recovery from depression (EPDS < 6) at 6 months. Secondary outcomes include measures of disability, parenting skills, maternal attitudes, health care utilization as well as infant physical and cognitive development comprehensively assessed using the Bayley’s Scales.

**Discussion:**

To the best of our knowledge, this is the largest randomized controlled trial of an intervention package delivered by community midwives in sub-Saharan Africa.

**Trial registration:**

Trial is registered with the ISRTCN registry at isrtcn.com; Trial number ISRCTN60041127. Date of registration is 15/05/2013.

## Background

Depression is one of the most common disorders in adult life with adverse consequences for health and social wellbeing [1]. More than 13 % of the global burden of disease is due to neuropsychiatric disorders, with depression being the leading cause of the burden [2, 3]. Epidemiological studies conducted in Nigeria find 10–20 % of consecutive attendees in primary care have depression [4, 5]. Depression is particularly common amongst women of child bearing age. One systematic review estimated the point prevalence of major and minor depression to be between 8.5 and 11 % during pregnancy and between 9.7 and 12.9 % postnatally [6]. In Nigeria, rates of up to 10–30 % have been reported amongst perinatal women [7–11].

Perinatal depression, defined as non-psychotic depressive episode often beginning during pregnancy or the postnatal period, is a substantial public health problem, not only because it is common but also because it is associated with long term adverse consequences on infant development and mother-child interactions [12]. Maternal depression is a risk factor for pre-term birth and low birth weight, infant under-nutrition and stunting as well as higher rates of diarrhoeal diseases [13–17]. Infants of depressed mothers also show poorer infant development as assessed using standardized measures, poorer interpersonal functioning, insecure attachment and elevated rates of emotional and behavioural problems [16, 18–20].

Worldwide, only a minority of depressed persons get the care they need [21]. Depressed women, especially those in the perinatal period, are particularly less likely to have their mental health needs met. It has been estimated that less than 50 % of cases of postnatal depression are detected by primary health care professionals in routine clinical practice [22]. This estimate was made for settings with much better resourced health care systems than are found in low and middle income countries (LMIC). It is very likely that a much smaller proportion of cases are identified and treated in Nigeria and other LMIC. Hence there is a huge level of unmet need for service in LMIC. For example, a previous study in Nigeria found that about four out of five persons with severe mental disorders, particularly depression, had received no treatment in the previous year and that, among those who did, only about 10 % had received what could be described as minimally adequate treatment [21]. The extent of the treatment gaps in LMIC reflects the extreme paucity of resources, both human and material. In sub-Saharan Africa, there is less than one psychiatrist to one million population and the few available specialists are based in urban areas [23]. Less than 2 % of the national budget goes to health and, within this, mental health service is hardly featured [23]. In addressing these gaps, it is now commonly agreed that the most effective way to bring health care to more people in need is through the integration of mental health services into routine primary care and maternal care so that non-physician health care providers at primary health clinics or dispensary level deliver the bulk of essential mental health service under the supervision and support of physicians or psychiatric nurses at district/local government level, who are themselves supported by more highly trained mental health specialists at regional/state levels [24].

Evidence from systematic reviews shows that perinatal depression can, in most instances, be effectively managed with psychological treatments [25, 26]. Even though much of this evidence has come from studies in high-income countries, previous studies in some LMIC have also shown that psychological interventions can be delivered by non-specialist health workers, such as community health workers (CHWs), including community midwives (CMWs) [27–29]. The results of these studies suggest that there is a need for training of the CMWs to deliver the intervention in a way that is sustainable within their day-to-day practice and that close supervision and support are available to these providers.

There is now considerable evidence in support of stepped care approaches to expanding mental health services [30]. In this model, non-physician primary care providers deliver the bulk of essential mental health service under the supervision and support of physicians and of more highly trained mental health specialists, if these are available. The essential ingredients to scale up mental health services to perinatal women are therefore generally agreed upon. However, considerable challenges remain to translate this knowledge into practice in diverse settings. This is especially so in regard to building an integrated treatment system out of the available resources in LMIC.

## Methods/Design

### Aim of the study

The primary aim of this study is to test the effectiveness and cost-effectiveness of a stepped care intervention package for perinatal depression delivered by community midwives in primary maternal care, supported by physicians and mental health specialists through the use of mobile phones, compared to usual care in a randomized controlled trial.

The primary hypothesis is that the intervention package will be more effective at alleviating depression at 6 months after childbirth compared to care as usual. The secondary hypotheses are that: 1) the intervention package will be more cost-effective; 2) will lead to better infant outcomes (in regard to cognition and growth) at 12 months; 3) will be associated with better parental skills of mothers; and 4) will lead to less experience of perceived stigma.

### Design

This study was a two-arm parallel cluster randomized controlled trial comparing an intervention package for maternal depression consisting mainly of psychological interventions (psychoeducation, problem solving treatment and parenting skills) with care as usual. The unit of randomization was the primary maternal care clinic; interventions and outcome measures were however administered on individual participants. As the intervention was designed to be delivered by clinic staff, the cluster randomized design was chosen in order to reduce the potential risk of contamination within clinics.

### Setting

The study was conducted in Oyo State, Nigeria. The state has 33 local government areas (LGA). All 137 maternal and child care clinics (MCCs) in nine LGAs selected for the study (five rural and four urban) were assessed for eligibility to participate. MCCs are the main source of care for most low-income perinatal women and are therefore the appropriate place to test an intervention package that is designed to make a major impact on the delivery of care to this population. Maternal and child health services in these clinics are largely delivered by non-physician primary care providers and midwives, who have been trained as nurses, community health officers and community health extension workers. Each of these categories of providers has a minimum of 2–3 years post-secondary education and are certified by their respective boards. Supervision for all the clinics in each LGA is provided by one general practitioner employed by the government and designated as the Primary Health Care Coordinator for the Local Government.

We used both urban and rural areas in order to capture the diversity of socioeconomic profile of the country and because access to medical treatment differs substantially between these two types of setting, thus making the demonstration of the utility of our program potentially more generalizable.

Eligible clinics were those that offer full maternal and child health services (*n* = 49). The study procedures were described to the supervising physicians in each of the LGAs and to the facility managers in each of the eligible clinics. Only clinics providing explicit consent to participate were randomized into the trial. Of the 30 that did so, one was excluded because the staffing profile was too thin to permit for effective participation, thus leaving 29 included in the trial.

### Randomization

Eligible and consenting maternal care clinics were stratified by local government area and allocated to intervention or control arm using a computer-generated random number sequence. Allocation was conducted by one of the authors (AAM) using anonymous codes for clinics and LGAs provided by other members of the research team, in order to avoid any risk of selection bias.

### Ethics and research governance

The study was approved by the University of Ibadan/ University College Hospital Ibadan Ethical Review Committee and was conducted in strict compliance with the guidelines specified by the Committee. The conduct and progress of the study were monitored by three committees: 1) Trial Management Group: This was responsible for providing study oversight including giving operational direction to the study and monitoring progress. This Group, which had monthly teleconference and annual face-to-face meetings, included the investigators and senior researchers; 2) Trial Steering Committee: Its role was to monitor and supervise the implementation of the trial, ensure that there were no major deviations from protocol specifications, and offer advice to the investigators on the scientific conduct of the trial. The Committee was made up of three independent members and scheduled two annual meetings, one face-to-face and the other by teleconference. Additional teleconference meetings were called when information from the Principal Investigator required immediate advice; and 3) An independent Data Monitoring and Ethics Committee whose responsibility was to advise the TSC on ethical and safety issues, including whether such issues could affect the continuation of the study.

### Recruitment of participants and eligibility

Considering that allocation concealment and selection bias could be a problem in cluster randomized trials where all participants are not consented and recruited prior to allocation of clusters, consecutive attendees at the selected MCCs were invited to participate by trained research staff while waiting to see the midwife. They were briefly educated about perinatal depression and invited to take the screening interview. Those who agreed were screened with the Edinburgh Postnatal Depression Scale (EPDS).

Women who screened positive (EPDS score of 12 or greater) and were eligible for the study (see eligibility criteria below) were invited to participate in the study. Those who provided signed consent following a full description of the study were entered into the trial. Consenting patients were handed their EPDS score and told to see one of the trained CMWs in the clinic.

Women most commonly present for the first time for antenatal care early in the second trimester in these clinics. We aim for the findings of our study to be as widely generalizable as possible. We therefore have very limited exclusion criteria.

**Inclusion Criteria** (patients must satisfy all of the following to be considered for study entry)

- Women aged between 16 and 45 years.
- Gestational age of between 16 and 28 weeks.
- Must score 12 or more on the Edinburgh Postnatal Depression Scale, a validated cut-off score indicating depression of at least moderate intensity. This cut-off is supported by previous validation studies in Nigeria [8, 31].
- Confirmed DSM-IV diagnosis of depression using relevant questions from the Composite International Diagnostic Interview (CIDI) [32].
- Provide informed consent.

**Exclusion Criteria** (patients were excluded from study entry if any of the following was met)

- Immediate need for medical attention
- Actively suicidal
- Presence of bipolar or psychotic disorder
- Unlikely to be in the neighbourhood in the following 12 months

### Interventions

The intervention package for EXPONATE was an adaptation of an intervention for adults with depression which we had developed and piloted in our earlier study of a stepped care intervention for depression in primary care [33]. The intervention was pragmatic, fully manualised, and took account of specific issues relating to pregnancy, childbirth, marital relationships and parental roles. It incorporated the treatment specifications of the WHO Mental Health Gap Action Programme Intervention Guide (mhGAP-IG) as adapted for the health system of Nigeria [34]. The manual consists of a step-by-step description of psychoeducation, problem solving treatment, social network activation and parenting skills. The intervention was developed following formative research assessing help seeking and experience of care for perinatal depression and contextualization workshops with care providers. These activities enabled us to incorporate culturally relevant experiences, norms, and contexts into the psychological component of the intervention.

**Fig. 1:**
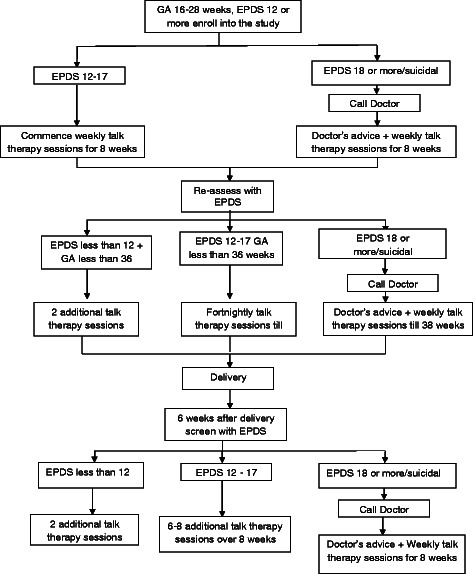
Treatment flow chart

The intervention package is designed to be delivered in three steps determined by the patient’s score on the EPDS, time since enrollment and gestational status. (See Fig. 1). All consenting and eligible participants with a score of 12 or greater on the EPDS in the intervention arm of the trial were enrolled into Step 1. Step 1 comprised 8 sessions of psychological interventions delivered weekly in the antenatal period. At the end of these first 8 sessions, patients whose gestational age was less than 36 weeks and who still had depression (indicated by an EPDS score of 12 or greater) continued with fortnightly psychological intervention sessions. Step 2 commenced 6 weeks after delivery during the mother’s routine first post-natal clinic visit. Patients were assessed by the CMW using the EPDS. Patients who scored below 12 received 4 fortnightly top-up sessions of the problem solving treatment. Those whose EPDS score was 12 and above received 8 weekly intervention sessions. At the completion of step 2, patients who still had EPDS scores of 12 or more proceeded to step 3. In step 3, patients had to be reviewed by the community physician with a view to initiating pharmacotherapy in addition to continuing with the psychological intervention or referral to specialist service.

Any patient in the intervention arm with a score of 18 or more or who had suicidal ideation at any time after entry into the trial was reviewed by the community physician who took a decision about the need for a psychiatric review/referral or prescription of medication in addition to the psychological intervention being delivered by the CMW. For patients who required medication, as assessed by the primary care physician, the first line was amitriptyline, which non-physician primary care providers in Nigeria are authorized to prescribe.

Each session of the psychological intervention lasted approximately 30–45 min. The CMWs were encouraged to schedule sessions outside of busy clinic hours and at times that were agreeable to both the patient and the CMW. This ensured that sessions could be conducted in strict privacy and with less likelihood of intrusion. The first session was dedicated mostly to familiarizing patients with the health care provider, and providing psychoeducation. In psychoeducation, the nature of the patients’ illness and the modality of treatment were explained in simple language using local expressions while avoiding the labeling of ‘mental illness/disorder’. Patients were encouraged to ask questions and were free to express their feelings. In addition, patients were provided with information booklets written in Yoruba and supported with pictorial representations of depression and the intervention modalities provided in the program. Psychoeducation was continued at each subsequent session.

Parenting skills and activity scheduling (behavioral activation) were introduced from the second session. In activity scheduling, patients worked with the CMW to schedule activities that were important or pleasurable to them. Patients were assisted to make a list of pleasurable activities and household chores that they had stopped doing on account of their depression; the CMW then worked with the patient to select at least one to be carried out over the next week. These activities were noted in the patients’ record book kept in the clinic as well as patients take-home card with ‘home work’ tasks. ‘Home work’ tasks from the preceding session were reviewed at the beginning of the next session. The parenting skills component was used to emphasize routine information provided to antenatal women (such as importance of routine antenatal visits, adequate nutrition and rest, preparation for delivery and signs that could indicate an emergency) as well as educate the woman on newborn care, infant nutrition and responsive parenting.

Problem solving treatment (PST) was introduced at the third session. We delivered a local adaptation of Problem Solving Treatment for Primary Care (PST-PC), which had earlier been used by us in studies in primary care. PST is an effective seven-step common sense talk therapy that aims at helping patients resolve troublesome problems that contribute to causing or prolonging the depressive episode [35, 36]. It involves working with the patient to identify and explore the problems that they are currently faced with and aiding the patient to develop and implement practicable solutions. The CMW always reviewed with patients their attempts at problem solving at the beginning of each session, praising their efforts and relating improvement in depression symptoms to their improved ability to deal with problems.

In the seventh session, using a problem solving approach, the CMW helped the patient to explore their social network with a view to selecting the most appropriate person to provide support for the patient in terms of caring for the new born, older children and household chores following delivery. Part of the patient’s take-home tasks for this session included contacting this caregiver. The last antenatal session was used to review the patients’ progress, emphasize on parenting skills learnt and encourage the patient to continue using the problem solving skills acquired in the course of the treatment sessions on their own. Psychological sessions conducted postnatally provided opportunities to revisit the issues discussed in the prenatal sessions, especially in light of arrival of the new baby, explore further the issues of social (and marital) support, and parenting skills.

### Control arm

Subjects who were recruited in the control clinics were also provided with their EPDS scores and advised to show these to their health care providers. Subjects in this arm were provided with enhanced usual care which included all services normally available in the clinics; including antidepressant medications, brief psychotherapeutic interventions, medical consultations, or external referral for specialty treatment. Although all these options were potentially part of usual care, in reality, unstructured counselling was often all a patient with recognized perinatal depression will receive. Structured psychotherapy, consultation with a doctor or referrals to a specialist were very rarely offered. Our usual care is “enhanced” because providers in the control arm received training on the recognition and management of depression based on the specifications of the mhGAP-IG prior to commencement of recruitment into the trial.

### Training

Prior to recruitment of patients, providers in the intervention arm received training on the recognition of depression, the delivery of the manualized intervention package, the procurement and documentation of support and supervision using mobile phones, and the use of the automated electronic support and documentation system. The training consisted of didactic lectures enhanced with clinical demonstrations and role playing over a 3-day period. They had a further 3-day top-up training about a month into the study to reinforce the skills previously acquired and to identify any difficulties they had encountered in administering the intervention.

Training of the providers in the control arm was conducted separately. They received a 2-day training on identification and standard treatment of depression. This training was based on the mhGAP-IG but without detailed PST training or guidelines and procedure for obtaining structured support and supervision from physicians. The CMWs from the control clinics were also provided with manuals providing general information about perinatal depression.

Lay research assistants were recruited and trained. The research assistants, all have at least a college degree, were trained to administer the outcome assessment instruments using the computer tablets provided. The research assistants had a 4-day training in the administration of the screening and outcome tools and conducted several trial live interviews before commencing field work. For the Bayley’s Scales of infant development, a Clinical Psychologist with vast experience in the use of the scale conducted an initial one-week training. During this training, the trainer gave didactic lectures on the use of the Scale and demonstration assessments of 2 infants. This was followed with supervised assessment of 2 infants by each of the 5 research assessors. Next, an inter-rater exercise with 20 infants was conducted with each of the assessors conducting 4 assessments that were independently rated by the others. Finally, a further paired assessment of 2 infants was interchangeably conducted by two assessors with one assessor conducting the assessment and the other observing and providing feedback afterwards.

### Monitoring and supervision

The components of and tasks for each treatment session as well as clinical decisions and steps were detailed in manuals and charts provided to the CMW and primary care physicians. To facilitate support and supervision to the CMW, we developed a technological support platform that incorporated a network of mobile telephones linked to an online record system hosted on a secure server. Mobile telephone lines were provided to each of the trained CMW from the intervention clinics and their supervisory community physicians and supervising psychiatrist. These mobile phone lines are linked in a closed user group network where calls within the network are free to facilitate consultation for difficult cases. The CMW were encouraged to call patients to remind them of their clinic appointments. Patients similarly received automated voice messages to reinforce key messages provided during sessions as well as remind them of clinic appointments.

### Process evaluation

The Trial Manager monitored adherence to protocol specification throughout the period of the intervention. This was done by way of regular qualitative as well as quantitative assessments. An assessment of fidelity in the delivery of the intervention was conducted by an independent rater who observed three pre-selected live sessions by each of the providers in the intervention arm using a scale developed for the purpose.

### Outcome measures

The outcome assessments, still on-going at the time of this report, are conducted mostly in the patients’ homes (except when patient prefers to have the assessment elsewhere) by the trained research staff. Outcome assessors have no involvement in delivering the intervention and are rotated between MCCs to collect outcome data. All consenting patients receive baseline assessment within 72 h of being screened and consenting to enter the trial. Follow-up assessments are conducted at 2 months following recruitment into the study and 3, 6, 9 and 12 months after childbirth. All scales are administered in their Yoruba versions, derived through standard iterative procedures which we have employed in previous studies.

### Primary outcome

The primary outcome is recovery from depression at 6 months postpartum (i.e. no longer having significant depressive symptoms as indicated by EPDS score less than 6).

### Secondary outcomes

- Score on the EPDS: in order to address the possibility that even though individuals may have recovered from a major depressive disorder, significant depressive symptoms may still persist. The EPDS is a 10-item screening instrument for depression. It has been validated and used in earlier studies of perinatal depression in Nigeria [11].
- Disability in the mother is assessed using the WHO Disability Assessment Scale [37], previously used by us [38];
- Parenting skills (measured as maternal attitude and adjustment to parenting) using the Maternal Adjustment and Maternal Attitudes Questionnaire (MAMAs), a widely used questionnaire with good acceptability to women and good reliability [39] as well as the Infant Toddler version of the Home Inventory for Measurement of the Environment (HOME-IT) [40]
- An adapted version of the experience of stigma by mothers with the 12-item Discrimination and Stigma Scale, a cross-culturally valid tool that has been previously used among Nigerian subjects [41]
- The Client Service Receipt Inventory-PND version [42], an adaptation of the Service Utilization Questionnaire (SUQ) which we have used in previous studies to assess health service utilization [43]
- Infant development: the child’s current level of cognitive, language, personal-social, and fine and gross motor development with an infant well-being questionnaire. This questionnaire was developed by us and it incorporates questions on child nutrition, achievement of developmental milestones, illnesses, immunization and measurements of the length/height and weight and administered at 3, 6, 9 and 12 months. At 12 months, a comprehensive assessment of motor and cognitive development is conducted using the Bayley Scales of Infant Development [44] which has been used previously among Nigerian subjects [45].

**Fig. 2:**
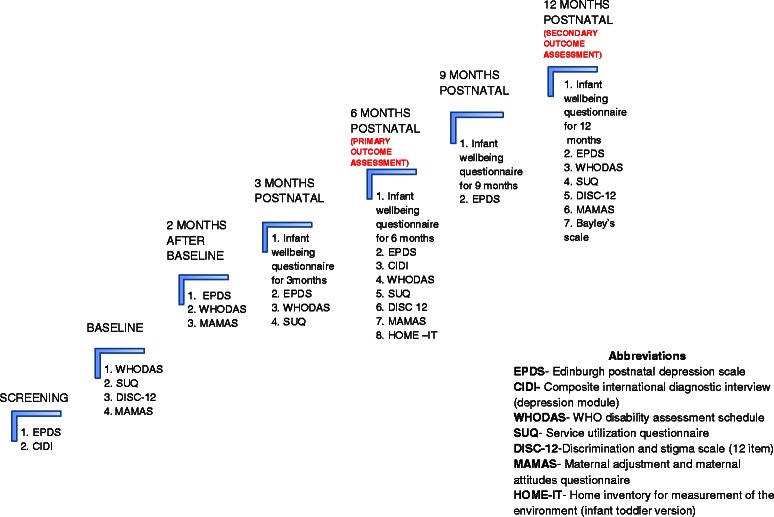
Timelines for outcome assessments

The times and components of all scheduled outcome assessments are shown in Fig. 2. Outcome data is collected from every participant not known to have died at the time of follow-up and who has not withdrawn consent, regardless of compliance with allocated treatment.

### Sample size

Experience from our previous studies in primary health care (PHC) as well as from the control arm of a Pakistani trial among women with perinatal depression suggests a recovery rate of about 45 % for major depression with no active treatment and about 75 % with treatment [29]. In a Chilean study of postnatal women with depression, using a 3-point reduction in baseline score of the depression assessment tool, remission rate was 57 % with no active treatment and 73 % with active treatment [28]. For our sample size estimation, we sought to detect an absolute difference of 15 percentage points (40 % recovery in control and 55 % in intervention groups respectively) at 6 months, a difference that we think is both plausible for this type of intervention and would promote changes in practice. In our pilot study among antenatal women, we observed an intra-cluster correlation coefficient (ICC) of 0.026 for EPDS scores. In our pilot study among general adults with depression, we were able to collect primary outcome at 6 months from 87.8 % of the participants. We expect a higher follow-up rate among perinatal women who generally are less likely to move. However, we will use a conservative 6-month follow-up rate of 85 % for our estimate. We also assume a 7 % infant death rate within the period. The uninflated sample size requires 186 per arm for analysis to detect a difference of 55 % vs 40 % (equivalent odds ratio = 1.8) with 80 % power at the two-sided 5 % alpha level. We originally aimed to recruit 50 individuals per MCC. With 43 individuals per cluster available for the primary analysis and an ICC of 0.026, the design effect is 2.09 giving a total number required for analysis of 778. We therefore aimed to recruit a total of 916 individuals from a total of 18 MCCs. As participant recruitment was slower than anticipated, in January 2014 we recruited and randomised a further 11 MCCs, giving a total of 29 in the study. Participant recruitment took place between July 2013 and October 2014. Outcome assessments are still ongoing and will continue till one year post-delivery assessment is concluded for the last recruited participant.

### Data analysis

A full statistical analysis plan will be developed before any data are analyzed. The analysis and presentation of the trial will be in accordance with CONSORT guidelines for cluster randomized trials [46, 47]. The primary approach for comparative analyses will be to analyze participants as randomized without imputation of missing data, and with due emphasis placed on confidence intervals for the between-arm comparisons. We will use descriptive statistics to assess balance between the trial arms at baseline for both clinic and individual participant characteristics. In order to take appropriate account of the hierarchical nature of the data, we will use multivariable mixed effects regression models to estimate recovery from depression at 6 months for intervention group versus control, adjusting for baseline depression and LGA as a stratification variable. In a secondary analysis, we will further adjust for any variables that were imbalanced between trial arms at baseline. These analyses will be repeated for secondary outcomes. We will conduct sensitivity analyses to assess the potential effect of missing data, and will investigate the effect of adherence with the intervention. We will investigate whether between-group differences vary over time using data from all follow-up visits in repeated measures analyses.

We will investigate whether there is any differential effect of the intervention according to baseline symptom severity (EPDS score <16, ≥16) by including appropriate interaction terms in the primary regression model. Since the trial is powered to detect overall differences between groups rather than interactions of this kind, the results will be interpreted with due caution.

Data analysis will be conducted once all follow-up is complete. There are no planned interim analyses.

## Discussion

EXPONATE is designed to provide empirical data that will improve our understanding of the management of perinatal depression in resource-constrained settings. Its findings should inform the development of programs to scale-up feasible and effective intervention through primary maternal care service.

## Abbreviations

LMIC: Low and middle income countries
EPDS: Edinburgh postnatal depression scale
CHW: Community health worker
CMW: Community midwives
LGA: Local government area
MCC: Maternal and child care clinic
MHGAP-IG: WHO mental health gap action programme intervention guide
PST: Problem solving treatment
PHC: Primary health care
